# Transcriptome sequencing and screening of genes related to sex determination of *Trichosanthes kirilowii* Maxim

**DOI:** 10.1371/journal.pone.0239230

**Published:** 2020-10-15

**Authors:** Xiuqin Hu, Zhenyang Liao, Bo Zhang, JingJing Yue, Zhen Wang, Xin Jie, Juan Liu

**Affiliations:** 1 Lunan Engineering Technology Research Center for the Development of Traditional Chinese Medicine Resources of Shandong Province, School of Pharmacy, LinYi University, Shandong, China; 2 FAFU and UIUC-SIB Joint Center for Genomics and Biotechnology, Fujian Provincial Key Laboratory of Haixia Applied Plant Systems Biology, Key Laboratory of Genetics, Breeding and Multiple Utilization of Corps, Ministry of Education, Fujian Agriculture and Forestry University, Fuzhou, Fujian, China; 3 Key Laboratory of Plant Germplasm Enhancement and Specialty Agriculture, Wuhan Botanical Garden, Chinese Academy of Sciences, Wuhan, China; USDA Agricultural Research Service, UNITED STATES

## Abstract

*Trichosanthes kirilowii* Maxim. (*TK*) is a dioecious plant in the Cucurbitaceae for which different sexes have separate medicinal uses. In order to study the genes related to sex determination, transcriptome sequencing was performed on flower buds of male and female plants using the high-throughput sequencing technology. A total of 145,975 unigenes and 7110 DEGs were obtained. There were 6776 DEGs annotated to 1234 GO terms and enriched to 18 functional groups, including five biological processes related to sugar metabolism. KEGG pathway analysis indicated genes involved in hormone transduction, hormone synthesis and carbohydrate metabolism. Many DEGs of *TK* are involved in reproductive organ formation, hormone signal transduction and regulatory networks. Combining the results of GO, KEGG and qRT-PCR, 11 sex determining candidate genes of *TK* were selected, including *MYB80*, *MYB108*, *CER1*, *CBL9*, *ABCB19*, *SERK1*, *HSP81-3*, *ACS9*, *SEP3*, *AUX1* and *YUC6*. The results provide a foundation for the study of sex differentiation in *TK*.

## Introduction

*Trichosanthes kirilowii* Maxim. (namely *TK*) is a perennial climbing herb in the family Cucurbitaceae. Its fruit (fructus trichosanthis), seeds (semen trichosanthis), peel (trichosanthis pericarpium) and root (radix trichosanthis) are all commonly used as traditional Chinese medicines. Due to the large demand for medicinal products in the marketplace, there are many planting bases for *TK* in China. Among these, Changqing District of Jinan City, Shandong Province and the surrounding areas of Feicheng City, Shandong Province have a long history of producing excellent varieties and high-quality medicinal materials that are famous as genuine herbal medicines.

Dioecious plants play an important role in elucidating the mechanism of plant sex determination and evolution, especially plants in the Cucurbitaceae. The studies of sex identification are of great significance in both theory and practice. *TK* is dioecious and cross pollinated, and the tissues used for commercial medicinal usages were differ between two sexes. When harvesting seeds and fruits, a large number of female plants (with a small number of male plants) are required, and when harvesting roots, male plants are required. At present, *TK* can be propagated in two ways: vegetative propagation using rhizomes and sexual propagation using seeds. Although the plant sex can be controlled by rhizome propagation, the propagation coefficient is low, and large amounts of raw materials are consumed. Therefore, seed propagation is an economical and practical method of improving the planting efficiency and realizing large-scale cultivation. However, the problem with seed reproduction is that the proportions of male and female plants cannot be controlled. In the natural state, the ratio of male to female is about 7:3. Therefore, it is of great significance to identify the early sex of *TK* seedlings and to reveal the molecular mechanism of sex determination.

At present, the methods for sex identification of *TK* include plant appearance, chemical reagents, isoenzymes, protein electrophoresis and molecular markers. Plant sex difference arises from differences in gene expression. Isozymes and proteins are the products of gene expression, and specific gene expression produces specific isozymes or proteins. Therefore, Yu et al. and Li used PAGE to identify the early sex of *TK* and found certain differences between male and female strains in enzyme concentration and spectral bands [1, 2]. Karmakar et al. used total proteins of *TK* roots to identify plant sex and found a slightly sex differential band with a molecular weight of 19 KDa [3]. Qu et al.’s study of RAPD-SCAR had found that S 1200 primers can generate a 600-bp amplification band specifically in male *TK* [4]. Guo carried out isozyme electrophoretic analysis of *TK* leaves and found that the isozyme bands and enzyme contents from leaves of different sexes were different [5]. While it is generally assumed that sex expression is dominated by the formation and accumulation of flowering substances, the above studies have shown that sexual difference exist in *TK* at the seedling stage.

Extended to the Cucurbitaceae family, although many plants in the family are dioecious, only Concinia indica was demonstrated having sex chromosomes with an XX/XY sex determination system [6]. The sex of other Cucurbitaceae plants is controlled by few genes with no sex chromosome evolved. For example, sex differentiation of cucurbit was controlled by three genes, ACS11, ACS7 and WIP1 [7]. Studies of cucumber (Cucumis sativus Linn.) demonstrated that external hormones and environment affected the process of sex determination, and genes related to, ethylene synthesis and induction such as genes CsACS1G, CsACS11, CsACS2, CsACO2 and CsWIP1, were verified involved in sex regulation [8, 9]. Although more and more metabolic pathways and genes have been found involved in gender regulation, the regulatory mechanism is not clear [9]. As a Chinese traditional medicinal plant, TK was less known by scientists in other countries besides China, with no information of genome reference and transcriptome data and few EST sequences. Although Chinese researchers have done extensive exploratory work in this field and have obtained some basic results, however, it is difficult to identified sex determining genes via the existing sex linkage markers and to explore the molecular mechanism of sex determination. Therefore, the lack of genomic sequences has become a bottleneck in the study of sex differentiation of *TK*.

The sexual difference of *TK* mainly observed in the flower organs. In this study, we performed RNAseq of the flower buds from female and male plants of *TK* to analyzed the transcriptomic profiles in different sexes. The goals were to search for differential expression genes (DEGs), and to screen for the key genes related to sex differentiation in order to lay a foundation for revealing the sex differentiation mechanism of *TK* at the molecular level.

## Methods and materials

### Sample collection

*TK* is planted in the Hebao field planting base of Pinyin, Shandong province (116.45 E, 36.28 N). Samples were collected in July 2016, including flower buds of female and male plants (around 2 mm) [10]. Set up 3 biological replicates for a total of 6 samples(samples of female and male flower buds are named F1, F2, F3, M1, M2 and M3). After sampling, plant buds were wrapped with tin foil and placed into liquid nitrogen, then stored in -80°C refrigerator.

### RNA isolation and quality assessment

Total RNA of each sample was extracted by Tripure, and the concentration (optical density 260 nm/280 nm ratio) and quality (optical density 260 nm/230 nm ratio) were measured using an Aglient 2100 Bioanalyzer, with which RNA integrity (RIN) above 7.5 were used for library construction.

### cDNA library construction, quality control and Illumina sequencing

Approximately 10 μg total RNA of each sample was used to constructed RNA libraries by NEBNext® Ultra™ RNA Library Prep Kit from Illumina® following the recommended protocol. The constructed libraries were sequenced by Beijing Institute of Genomics (BIG) under Illumina HiSeq 2500 sequencing platform with 150 bp pair-ends. The raw sequencing data reported in this paper have been deposited in the Genome Sequence Archive in BIG Data Center (Nucleic Acids Res 2019), Chinese Academy of Sciences, under accession numbers CRA002313 (https://bigd.big.ac.cn/gsa).

### Raw sequencing processing and *de novo* assembly

FastQc was used to detect the raw RNA reads and remove the joint sequences. The clean reads were obtained after removing connectors and low-quality reads (Q-value<10 or reads containing more than 5% ambiguous ‘N’ bases by trimmomatic (-l 5 -q 0.5 -n 0.1)). The clean reads were used to assembly using the Trinity (v.2.0.6), with a minimum contig length cutoff of 150 and a minimum k-mer size of 3 [11].

Over loop was used to splice the contig and unigene fragments of clean reads to obtain the unigenes. Finally, a BUSCO (Benchmarking Universal Single-Copy Orthologs) analysis was performed using BUSCO v.2.0 with default parameters to evaluate the completeness of total gene annotated in this study [12].

### Screening of differentially expressed genes

Cuffdiff of Cufflinks software (http://cole-trapnell-lab.github.io/cufflinks/) [13] was used to analyze the differences in gene expression levels in each group to identify the DEGs (differentially expressed genes). Cuffdiff uses non-parametric statistical methods to estimate the mean and variance of FPKM (expected number of fragments per kilobase of transcript sequence per millions of base pairs sequenced) values in different samples based on annotation files and identifies selected transcripts with significant differences in expression between samples through *t* tests.

### Verification of DEGs

6 differentially expressed genes were randomly selected and verified by quantitative real time PCR (qRT-PCR), to verify the consistency of expression patterns with RNA sequsing. For each sample, the PrimeScript First Strand cDNA Synthesis Kit with 1μg total mRNA (Takara, Dalian, China) was used to reverse-transcribe the mRNA into the first strand of cDNA, and the quality was measured by 1.5% agarose gel electrophoresis. We designed qPCR primers for specific genes using the Primer 5 software (https://primer-premier-5.software.informer.com/). The total volume of the quantitative PCR reaction system is 20 μL, including 10 μL of 2 × SYBR® Green I Master Mix (Takara, Dalian, China), each with 0.4 μmol/L of forward and reverse primers, 1 μL of cDNA template diluted ten-fold, and the final supplement ddH_2_O to 20 μL. The amplification was carried out with the following cycling programme: 30 s at 94°C, 40 cycles of denaturation at 95°C for 5 s, annealing at 55°C for 15 s, and extension at 72°C for 15 s on a ABI 7500 fast Real-Time PCR machine. A melting curve analysis was completed immediately after the qPCR. 18S rRNA was selected as the reference gene [14]. The relative expression level was calculated with the 2^−ΔΔCt^ method [15]. Three biological replicates and three technical replicates were performed for each of the analyzed genes.

### Functional annotation and classification

Through BLAST [16] comparison software (https://blast.ncbi.nlm.nih.gov/Blast.cgi), the unigenes sequences were compared with the protein databases NR (NCBI non-redundant protein sequences, http://www.ncbi.nlm.nih.gov/) [17] and KEGG (Kyoto Encyclopedia of Genes and Genomes, http://www.genome.ad.jp/kegg/kegg2.html) [18]. Classification information and gene function annotation were carried out by BLASTx (E-value ≤1.0E-05). In order to reflect the expression of sex difference genes more accurately, the GO (Gene Ontology, http://www.geneontology.org/) [19] function and KEGG pathway significance enrichment analyses were carried out to determine the main biological functions and the main metabolic pathways that the genes were involved in. GO enrichment analysis was performed on DEGs using the SEA tool of agriGo [20] software (http://bioinfo.cau.edu.cn/agriGO/), and the *P* values were statistically analyzed and corrected (FDR ≤ 0.05) using Fisher's exact test and the Bonferroni correction method. The KEGG pathway enrichment analysis uses KOBAS (E-value ≤1.0E-05)(KEGG Orthology-based Annotation System, http://kobas.cbi.pku.edu.cn/home.do) [21], where the calculation principle is the same as in the GO function enrichment analysis. To control the false positive rate, BH (Benjamini and Hochberg's test) [22] was used for multiple tests with P = 0.05. A KEGG pathway meeting the above conditions was defined as a significantly enriched pathway.

## Results and analysis

### Transcriptome sequencing and *de novo* assembly

We obtained 17,619,567 and 16,699,544 high-quality reads from the female and male libraries respectively. The effective detection rate of each library was above 90%. For species without reference genomes, *de novo* assembly is the most commonly used technique, and thus Trinity software (https://trinitysys.fm.alibaba.com/) was used to assemble the sequencing data. In all, 145,975 unigene fragments were obtained after redundancy removed. The assembly results are shown in Table 1. Among the resulting fragments, 0–400 nt had 71010 fragments; 400−800 nt had 44107 fragments; 800−2000 nt had 23331 fragments; 2000–4000 nt had 6648 fragments and there were 879 fragments ≥ 4000 nt. The completeness assessment result showed that 48.6% of BUSCO genes were “a single-copy”, 47.0% were “complete and duplicated” and 2.8% were “fragmented”, while the remaining 1.6% were “missing”, suggesting a good transcriptome assembly (Table 2).

**Table 1:** Length distribution of assembled unigenes from *TK*.

Length (bp)	Unigenes	Proportion (%)
**0–400**	71010	48.6
**400–800**	44107	30.2
**800–2000**	23331	16
**2000–4000**	6648	4.6
**>4000**	879	0.6

**Table 2:** BUSCO assembly evaluation results.

Sample	Complete and Single-copy	Complete and duplicated	Fragmented	Missing
**F1**	150	141	7	5
**M2**	154	135	8	6
**F3**	138	148	12	5
**M1**	136	154	9	4
**F2**	153	140	6	4
**M3**	153	137	8	5

### Identification and analysis of DEGs

We divide the genes into three categories according to the expression of FPKM value. FPKM ≥ 10 was set as a highly expressed gene, 2 ≤ FPKM < 10 as a medium expression gene, and FPKM < 2 as a low expression gene. According to the FPKM standard, we calculated the gene expression of flower bud samples as follows: the number of low, medium and high expression genes in female flower buds were 2491, 1924; and 1165 respectively; whilst the number of low, medium and high expression genes in male flower buds were 1588, 2463, and 1530. There were fewer low expression genes in male flower buds than in female flower buds, but more genes were expressed in male flower buds than in female plants. There were few differences in the medium expression genes of male and female flower buds, but there was a large difference in the number of high expression genes. Then, we used Cuffdiff to calculate the significance of differential gene expression, and set fold change > 2 or < 0.5, *P* < 0.01 as the criteria for identifying DEGs. We compared the gene expression in female relative to male samples, and defined the up-regulated genes in female flower buds as up-regulated genes. The number of DEGs in flower buds was 5580; the number of up-regulated genes was 3104, and the number of down-regulated genes was 2476.

### qRT-PCR validation of DEGs

**Fig 1:**
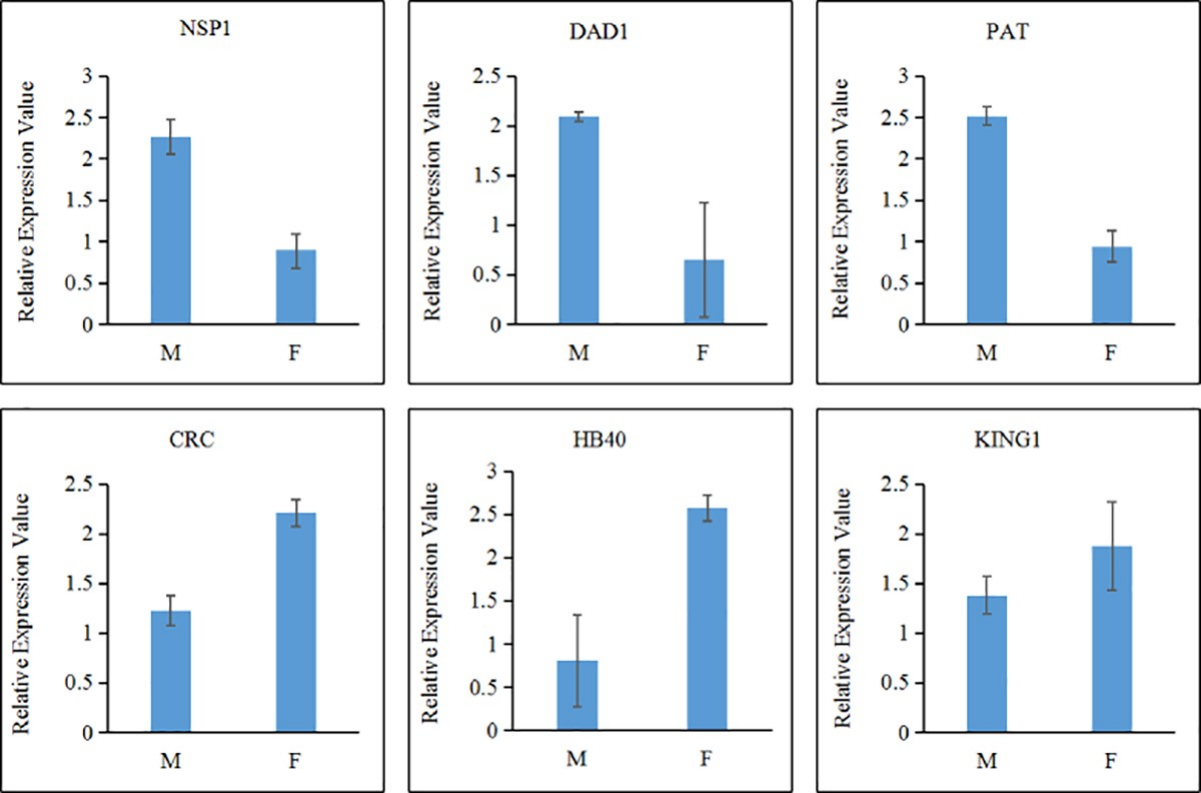
qRT-PCR validation of DEGS.

In order to confirm the results of Illumina sequencing, we randomly select 6 candidate genes and verified the expression of differentially expressed genes in male and female by using real-time PCR. The 6 candidate genes include 3 genes with high expression in the female libraries and 3 genes with high expression in the male libraries. The primer sequences of references genes and 6 selected genes are listed in Table 3. The expression trends of 6 genes in all samples are basically consistent with the expression trends obtained by transcriptome sequencing (Fig 1).

**Table 3:** Details of oligonucleotide primers used for qRT-PCR.

Gene Name	Annotation	Forward primers	Reverse primers
		(5’-3’)	(5’-3’)
***NSP1***	structural constituent of nuclear pore	CAACACAAAAAAAAGTAAA	TCAAAATGGGGTATGGAAA
***DAD1***	hydrolase activity	CAGTAGTGGCATTTAGAGG	ACAGTGACAAAGAGGGTGG
***PAT***	hydrolase activity, acting on ester bonds	ATCCAGAACAAAGGAAACC	CTACAATCCACTGAGCCAA
***HB40***	homeobox-leucine zipper protein	CAACACCAACTCCCCTCAA	CGTCGGCTTATTTCCCTCC
***CRC***	multicellular organism development	CAGAAACCGACCCACCAGC	TTTTTGAGCACAAGACCCC
***KING1***	5'-AMP-activated protein kinase, regulatory gamma subunit	GGCAACGACAGAGGAGAGT	AGGAAAAGCAGAAACAGGG

### Functional annotation and classification of DEGs

**Fig 2:**
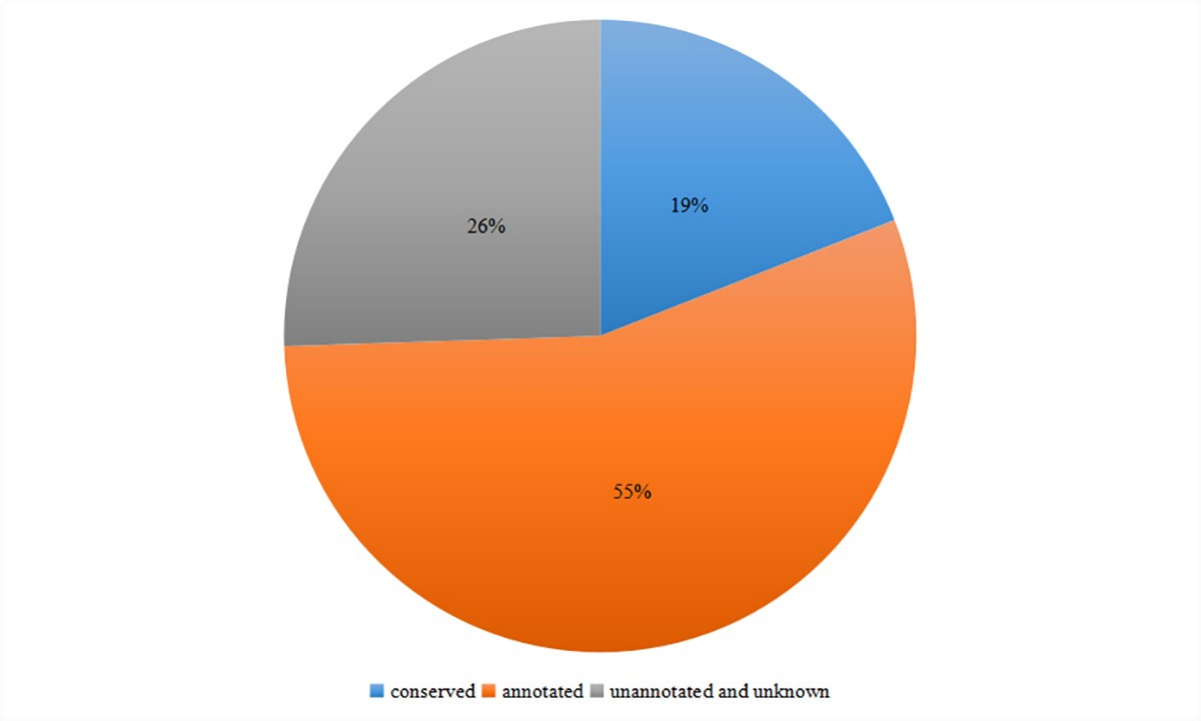
Functional annotation of the differential expression unigenes of *TK* in the nonredundant databases.

**Fig 3:**
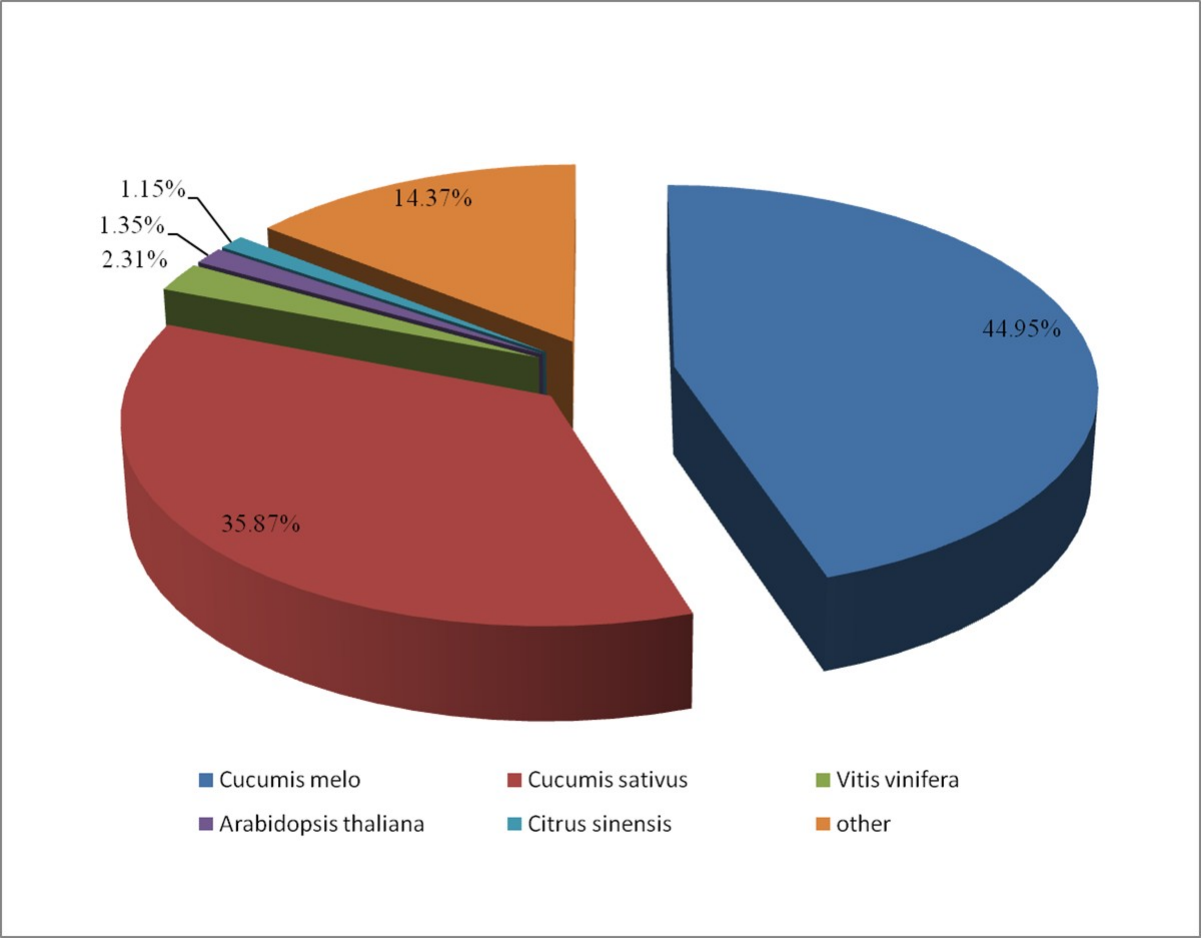
The species distribution of BLAST hits for each unigene in the Nr database.

All the differentially expressed sequences were submitted to NCBI for BLASTn comparison. A total of 5303 unigenes were annotated with the NR databases. Of those unigenes, 74% (3924) obtained homologous genes or obtained gene notes, and 26% (1379) had no homologous sequences or were position genes without functional annotation, as shown in Fig 2. Among the species that unigenes matched in the NR database, cucumber accounted for the highest proportion (15184, 44.95%), followed by *Cucumis sativus* (12117, 35.87%), *Vitis vinifera* (779, 2.31%), Arabidopsis (455, 1.35%), *Citrus sinensis* (387, 1.15%), *Cucumis melo* subsp. Melo (384, 1.14%), and other species (15.52%); 82% of the genes were annotated to Cucurbitaceae (Fig 3).

### Analysis and functional classification of DEGs

**Fig 4:**
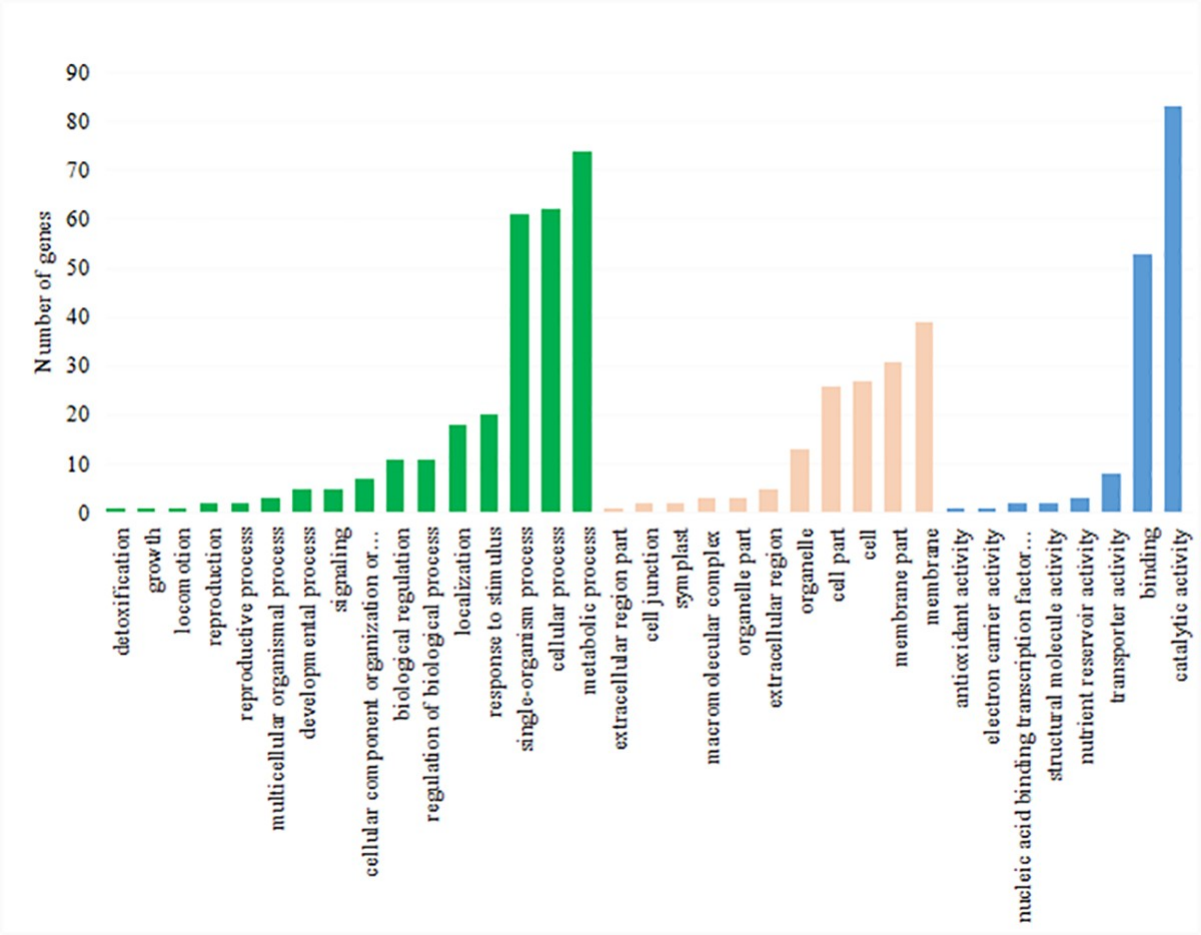
Gene ontology categories of the assembled unigenes from the *TK* unigenes were assigned to three categories: Cellular components, molecular functions and biological processes.

In order to further clarify the function of differentially expressed genes, the selected DEGs were analyzed with Blast2GO. The software comprehensively considers the similarity of target sequences and alignment sequences, GO item source reliability, and the structure of a GO directed acyclic graph, and extracts the qualified GO functional items in the mapping process (GO terms) annotated to the target protein (DEG protein). In this study, 6776 DEGs annotated 1234 GO items, including cell components (133), molecular functions (353) and biological processes (748). All the matched gene sequences were further enriched into 46 functional categories, among which the functional groups of membrane part, membrane, binding, catalytic activity, cellular process and metallic process contained more unigenes, while biological adhesions, location, protein binding, growth, extractor region part, rural reservoir activity and immune system contained fewer unigenes (Fig 4). Then according to the GO annotation information of significantly differentially expressed genes, we further analyzed the significance of enrichment and calculated *P* values by Fisher's exact test (FET). If FDR ≤ 0.05 and FDR ≤ 0.01, we assumed that there was significant enrichment or extremely significant enrichment of this GO function. The differentially expressed genes were enriched in 18 functional groups. These genes included the cell wall polysaccharide metabolic process (GO:0010383), hemicellulose metabolic process (GO:0010410), xyloglucan metabolic process (GO:0010411), hydrolase activity, acting on glycosyl bonds (GO:0016798), and xyloglucan: xyloglucosyl transferase activity (GO:0016762). The complete results are listed in Table 4.

**Table 4:** Significant enrichment of differentially expressed genes by GO.

GO term	description	*P*-value	Number in input/Ref
GO:0006928	movement of cell or subcellular component	0.00186	20 / 62
GO:0007017	microtubule−based process	0.01902	29 / 125
GO:0007018	microtubule-based movement.	0.00186	19 / 61
GO:0010383	cell wall polysaccharide metabolic process	0.00318	15 / 42
GO:0010410	hemicellulose metabolic process	0.00107	15 / 39
GO:0010411	xyloglucan metabolic process	2.5e−05	14 / 27
GO:0031224	intrinsic component of membrane	0.02239	448 / 4051
GO:0030312	external encapsulating structure	0.00113	40 / 195
GO:0016021	integral component of membrane	0.03884	444 / 4031
GO:0005618	cell wall	0.00113	39 / 193
GO:0044877	macromolecular complex binding	0.02069	32 / 158
GO:0016798	hydrolase activity, acting on glycosyl bonds	0.00025	61 / 328
GO:0032403	protein complex binding	0.00053	28 / 109
GO:0015631	tubulin binding	0.00498	23 / 90
GO:0016762	xyloglucan:xyloglucosyl transferase activity	2.04e−06	14 / 24
GO:0008017	microtubule binding	0.00114	23 / 83
GO:0003774	motor activity	0.00593	20 / 73
GO:0003777	microtubule motor activity	0.00122	19 / 61

**Fig 5:**
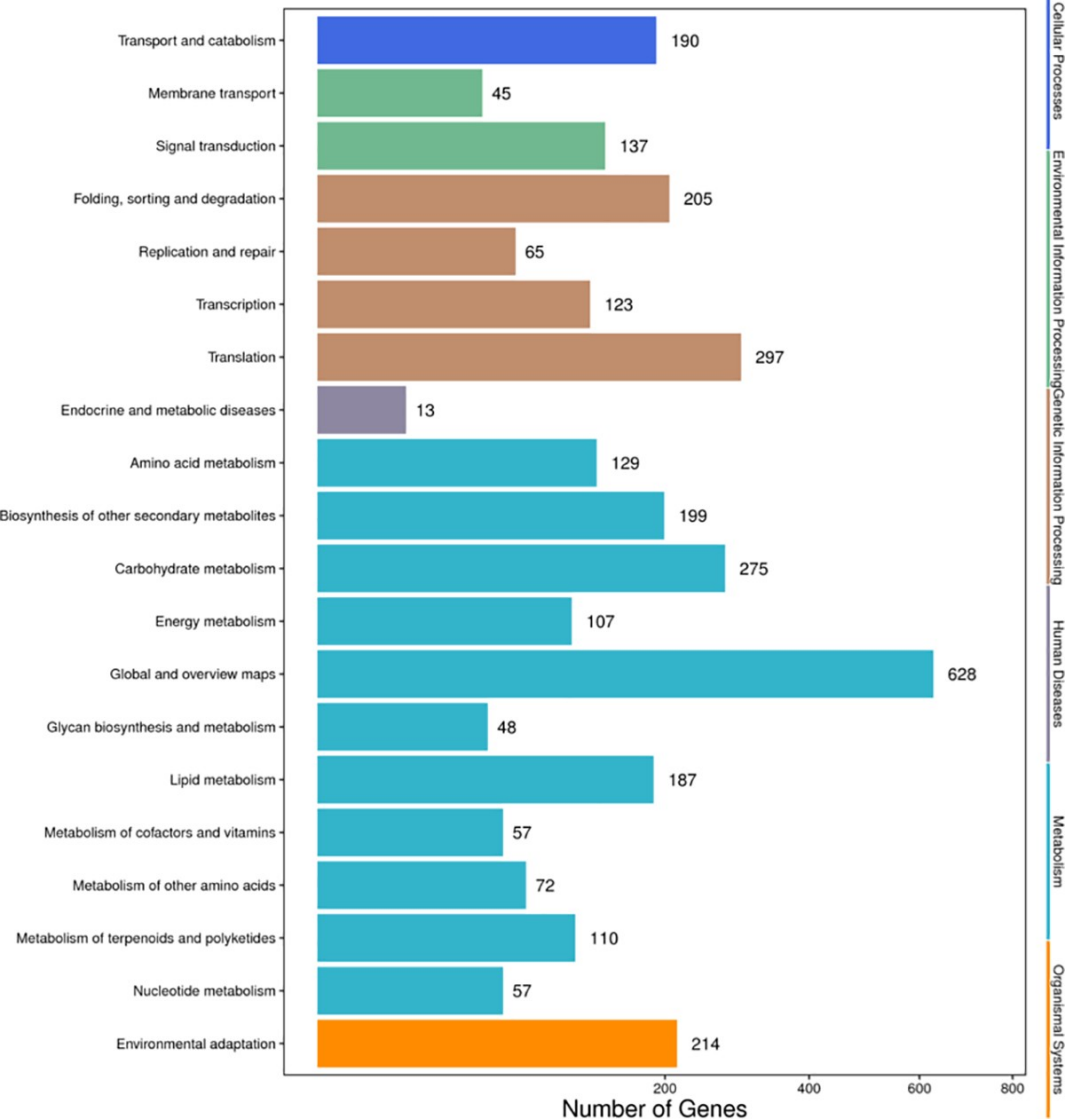
Distribution of Kyoto Encyclopedia of Gene and Genomes (KEGG) pathways in *TK*.

In order to further elaborate the biochemical pathways expressed by differential expression genes, KOBAS was used to compare the differential expression genes to the plant KEGG database, and an *E* value < 10^−5^ was set to identify the possible biological pathways. A total of 2286 different genes were located in 131 pathways, as shown in Fig 5. The pathways with more genes included global and overview maps, translation, carbohydrate metadata, environmental adaptation, folding, sorting and graduation, while endocrine and metallic diseases only had one gene. Twenty pathways were significant (*P* < 0.05; Table 5).

**Table 5:** Significant difference enrichment pathway by KEGGscreening of ge*n*es related to sex differentiation.

No.	Pathway	KO ID	*P*-Value
**1**	Linoleic acid metabolism	Ko 00591	0.00000
**2**	Phenylpropanoid biosynthesis	Ko 00940	0.00000
**3**	Stilbenoid, diarylheptanoid and gingerol biosynthesis	Ko 00945	0.00000
**4**	Flavonoid biosynthesis	Ko 00941	0.00000
**5**	Biosynthesis of secondary metabolites	Ko 01110	0.00003
**6**	Isoflavonoid biosynthesis	Ko 00943	0.00010
**7**	Plant hormone signal transduction	Ko 04075	0.00013
**8**	Limonene and pinene degradation	Ko 00903	0.00047
**9**	Cyanoamino acid metabolism	Ko 00460	0.00132
**10**	Plant-pathogen interaction	Ko 04626	0.00245
**11**	Pyruvate metabolism	Ko 00620	0.00460
**12**	Ribosome	Ko 03010	0.00485
**13**	Ascorbate and aldarate metabolism	Ko 00053	0.00788
**14**	Steroid biosynthesis	Ko 00100	0.01007
**15**	mRNA surveillance pathway	Ko 03015	0.01347
**16**	Circadian rhythm—plant	Ko 04712	0.01441
**17**	RNA polymerase	Ko 03020	0.02521
**18**	Glycolysis / Gluconeogenesis	Ko 00010	0.02724
**19**	Caffeine metabolism	Ko 00232	0.04228
**20**	Pentose and glucuronate interconversions	Ko 00040	0.04994

**Fig 6:**
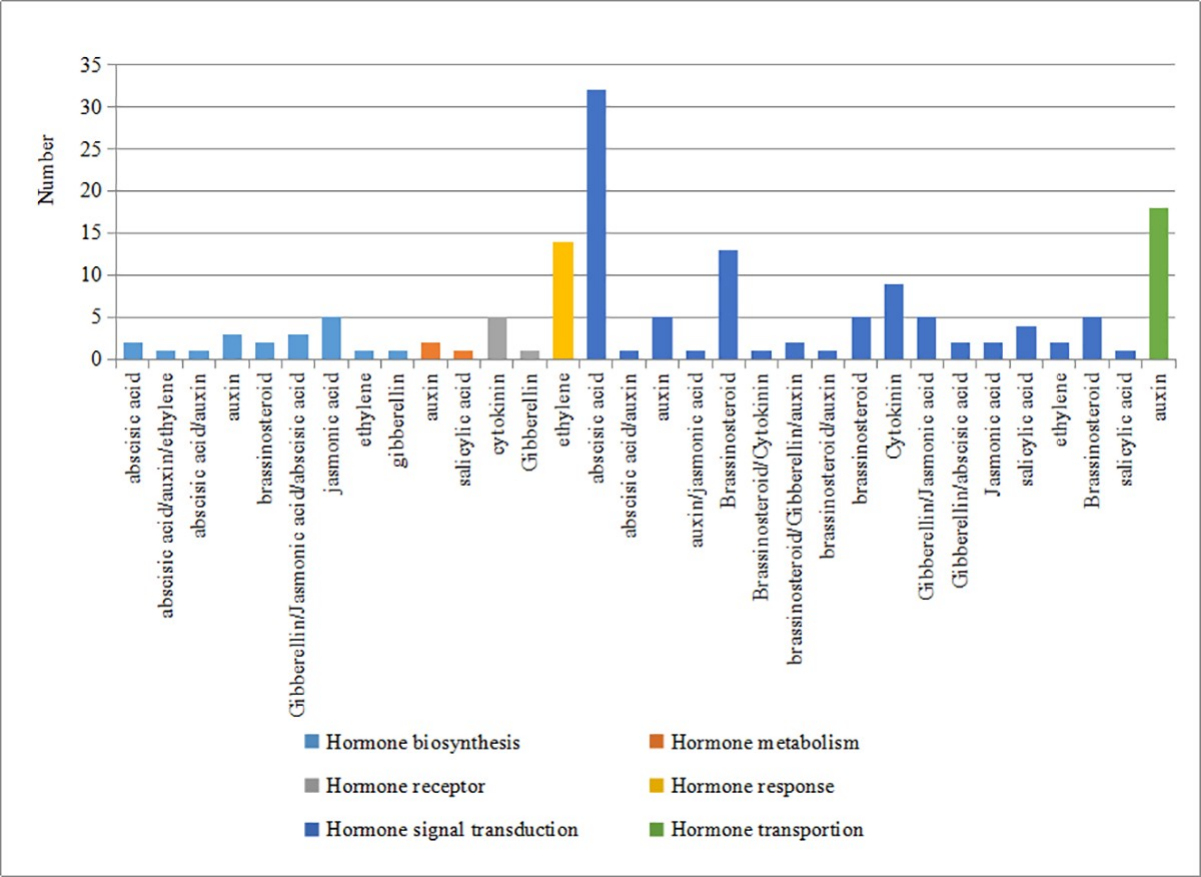
Hormone-related genes in TK.

Referring to the mechanism of sex differentiation in other Cucurbitaceae plants, hormone genes or genes induced by hormones are the main factors determining sex differentiation of *TK*. To date, except for *WIP1* orthologous genes, other sex-controlling genes, including *CsACS1G*, *CsACS2*, *CsACS11*, and *CsACO2* in cucumber, *CmACS7* and *CmACS11* in melon, *CitACS4*/*ClACS7* in watermelon, and *CpACS27A* in zucchini, have important roles in ethylene biosynthesis [9, 23]. Genes related to ethylene synthesis and genes induced by ethylene are particularly important [24–29]. We analyzed the DEGs related to hormones in *TK* by using blast P. A total of 7110 differential genes were compared with the Arabidopsis hormone database, and when the *E* value < 10^−6^ or the similarity ≥ 60%, we considered the two proteins to be homologous. According to this standard, we found 151 genes related to hormones from the DEGs, including 19 genes related to hormone synthesis, three genes related to hormone metabolism, six genes related to hormone receptors, 14 genes related to hormone response, 91 genes related to hormone signal transportation, and 18 genes related to hormone transportation (Fig 6). Combining literature studies on sex differentiation of Cucurbitaceae plants, GO, KEGG results and gene expression patterns of male and female plants, a total of 11 sex differentiation candidate genes were screened and compared with those in Arabidopsis (Table 6).

**Table 6:** Candidate genes related to sex differentiation of TK.

Gene_ID	Arabidopsis	Annotation	Gene symbol	Hormone	Function category	Identity
DN25049_c0_g1_i3	AT5G56110.1	myb domain protein 103	*MYB80*	Brassinosteroid	Hormone signal transduction	34.07%
DN29675_c0_g1_i1	AT3G06490.1	myb domain protein 108	*MYB108*	Jasmonic acid	Hormone signal transduction	15.74%
DN22459_c0_g1_i1	AT1G02205.1	Fatty acid hydroxylase superfamily	*CER1*	abscisic acid	Hormone signal transduction	22.46%
DN32496_c1_g2_i2	AT5G47100.1	calcineurin B-like protein 9*ACS9*	*CBL9*	abscisic acid	Hormone signal transduction	5.4%
DN36241_c0_g1_i2	AT3G28860.1	ATP binding cassette subfamily B19	*ABCB19*	auxin	Hormone transportion	28.53%
DN19445_c0_g1_i1	AT1G71830.1	somatic embryogenesis receptor-like kinase 1	*SERK1*	brassinosteroid	Hormone signal transduction	27.66%
DN34169_c1_g6_i5	AT5G56010.1	heat shock protein 81–3	*HSP81-3*	ethylene	Hormone response	3.75%
DN63827_c0_g1_i1	AT3G49700.1	1-aminocyclopropane-1-carboxylate synthase 9	*ACS9*	ethylene	Hormone biosynthesis	38.56%
DN13445_c0_g1_i1	AT1G24260.1	K-box region and MADS-box transcription factor family protein	*SEP3*	auxin	Hormone signal transduction	10.19%
DN1132_c0_g1_i1	AT2G38120.1	Transmembrane amino acid transporter family protein	*AUX1*	auxin	Hormone transportion	18.99%
DN72506_c0_g1_i1	AT5G25620.1	Flavin-binding monooxygenase family protein	*YUC6*	Cytokinin	Hormone signal transduction	18.05%

## Conclusion

Plant sex determination and differentiation have become a major focus of developmental genetic research in recent years. Compared with animals, plants have more variable sex determination patterns. Stamens and carpels require a large number of specific genes to participate in each development stage. Cucurbitaceae species are numerous, and their sexual systems are also variable. For example, the flower primordium of cucumbers is bisexual at first, and then the stamen or carpel stops development selectively, forming a unisexual flower [30]. However, the female flowers of *TK* are bisexual initially and the stamen development then stops, but the male flowers of *TK* are completely unisexual [10]. In addition, hormones and environmental factors can affect sexual development in the Cucurbitaceae, and ETH plays a major role [21]. For example, using ETH on monoecious watermelon plants will change all flowers into female flowers. In contrast, treatment of watermelon female plants with ETH inhibitors will lead to the occurrence of bisexual flowers. Consistent with the fact that ETH is a female hormone, watermelon gene A and cucumber gene M, as homologous genes, both encode the rate-limiting enzyme ACS during the ETH synthesis.

Our aim was to discover the sex determining genes in *TK*. The male and female flower buds of *TK* were selected as research materials according to the previous study [31]. After screening, 7110 differentially expressed genes were obtained, including 3694 up-regulated genes and 2942 down-regulated genes. Many genes involved in the formation of reproductive organs, hormone signal transduction and regulatory networks were indicated. In all, 6776 DEGs were annotated to 1234 GO items, and GO was enriched in 18 functional groups, including five biological processes related to carbohydrate metabolism This indicates that carbohydrate metabolism plays an important role in the sex differentiation of flower buds. Based on the KEGG pathway analysis, different genes of male and female plants were significantly enriched in steroid biosynthesis, RNA polymerase, glycolysis / glycogenesis, pentose and glycornate conversions; this suggest that hormones and sugars may be involved in the sex differentiation of *TK*. In view of the effect of hormones on the sex of Cucurbitaceae plants, we carefully analyzed the gene expression of hormone related genes? (HRGs). In total, 11 candidate genes for sex determination were selected from 151 hormone-related differential genes, including *MYB80*, *MYB108*, *CER1*, *CBL9*, *ABCB19*, *SERK1*, *HSP81-3*, *ACS9*, *SEP3*, *AUX1* and *YUC6*.

Among them, MYB transcription factor plays a very important role in higher plant anther development and pollen formation. *MYB80* encodes a MYB transcription factor that is essential for tapetal and pollen development [32–34]. MYB108 regulates late stages of stamen development and male fertility, and *MYB108* mutants exhibited reduced male fertility [35]. *CER1* gene involved in pollen fertility and it is responsible for pollen-pistil interaction in the self-compatible species Arabidopsis [36]. Ca^2+^ has been established as an important second messenger regulating pollen germination and tube growth. Related report has investigated the function of calcineurin B-like (CBL) Ca^2+^ sensor protein CBL9 in pollen germination and tube growth of Arabidopsis thaliana. And stable overexpression of *CBL9* strongly reduces pollen germination rates and alters pollen tube morphology [37]. There is evidence that the auxin transporter genes *ABCB19* are actively transcribed in both the early and late stages of stamen development. *ABCB19* mutant flowers have reduced stamen length as well as precocious pollen maturation and anther dehiscence [38]. The EMS1 (Excess Microsporocytes1) leucine-rich repeat receptor-like kinase plays a fundamental role in somatic and reproductive cell differentiation during early anther development in Arabidopsis. SERK1 and SERK2 may act as a co-receptor redundancy for EMS1, because the *SERK1 SERK2* double mutant phenocopies *EMS1*, although neither the *SERK1* nor *SERK2* single mutant shows detectable anther defects [39]. *HSP81-3* is a member of the heat shock protein 90 (HSP 90) gene family. It is expressed in all tissues, and is abundantly expressed in apical meristem, pollen and tapetum [40]. *ACS9* has been confirmed to be expressed in stigma [41]. In higher plants, the MADS-box genes encode a large family of transcription factors (TFs) involved in key developmental processes, most notably plant reproduction, flowering and floral organ development. *SEP3* is a member of the MADS TF family and it is important in determining flowering time as well as floral organ identity through the formation of multiprotein complexes with other MADS-family TFs [42]. In Arabidopsis, targeted auxin distribution is necessary for the morphogenesis and adaptive response of its organs, which involves the prototypical auxin influx facilitator *AUX1* and its *LIKE-AUX1 (LAX)* homologs. Report has analyzed and studied the *AUX1* homolog *BdAUX1* of *Brachypodium distachyon* (Brachypodium), which proves that *BdAUX1* is essential for the development of Brachypodium. *BdAUX1* loss-of-function mutants are dwarfs with aberrant flower development, and consequently infertile [43]. *YUC6* gene is involved in auxin synthesis during stamen development, and auxin can ensure correct and coordinated pollen maturation, anther dehiscence and filament elongation. The expression of these candidate genes was quantified by using male and female flower buds of different development lengths.The results showed that the expression of each candidate gene was significantly different between male and female plants, which was basically consistent with previous literature reports; however, the expression of myb80 in the female flower buds of TK showed an obvious trend of increasing at first and then decreasing. According to the results of paraffin section and expression trend of flower buds, we speculated that myb80 might be related to stamen abortion in female flower buds.Of course, these genes have only been confirmed to be related to sex differentiation in Arabidopsis or other plants, and whether they have the same role in *TK* needs further verification. The research results will be the basis for the research on the gender differentiation mechanism of *TK*.
